# The Assembly of Alginate–Chitosan–Xanthine Hydrogel and *Halomonas* Improves Soil Quality of Protected Crop Growth Under Saline–Alkali Stress

**DOI:** 10.3390/molecules31183215

**Published:** 2026-09-11

**Authors:** Rou Liu, Zirun Zhao, Guangbo Feng, Jiawen Yu, Xiaoxiang Zhou, Zijia Zhao, Danni Wang, Mingchun Li, Qilin Yu

**Affiliations:** National Key Laboratory of Intelligent Tracking and Forecasting for Infectious Diseases, College of Life Sciences, Nankai University, Tianjin 300071, China; 2120241635@mail.nankai.edu.cn (R.L.); 1120240822@mail.nankai.edu.cn (Z.Z.); guangbo@mail.nankai.edu.cn (G.F.); 2120241539@mail.nankai.edu.cn (J.Y.); 2120251720@mail.nankai.edu.cn (X.Z.); 2120251716@mail.nankai.edu.cn (Z.Z.); 2120251548@mail.nankai.edu.cn (D.W.); limchun@mail.nankai.edu.cn (M.L.)

**Keywords:** hydrogel, saline–alkali stress, protected agriculture, microbial inoculant, *Halomonas*

## Abstract

Soil saline–alkali stress has become a great risk, leading to significant decline in crop yields, especially in protected agriculture that is frequently threatened by excessive chemical fertilization. There is an urgent need to develop friendly strategies for improving the quality of saline–alkali soil. Microbial inoculants are promising agents in attenuating soil stress, but their colonization ability in the crop rhizosphere is often limited. To improve the efficiency of microbial inoculants, this study constructed the assembly of a salt-tolerant bacterium *Halomonas* (Homs) and a hydrogel composed of sodium alginate, chitosan and xanthine with the assistance of artificial Homs-binding protein (Schx). The effect of this Homs + Schx assembly on the growth of crops and the rhizosphere microecology under saline–alkali stress was systematically evaluated by pot experiments. The results indicate that the application of Homs + Schx enhanced the Shannon index of the rhizosphere bacterial community and increased the relative abundance of key bacterial genera related to biofilm formation and nitrogen cycling (e.g., *Curvibacter* and *Nitrospira*). Furthermore, Homs + Schx enhanced the activity of soil urease, peroxidase, and sucrase, reducing the sodium ion content, increasing the potassium ion content, and effectively lowering the soil pH and salt contents. Physiologically, this treatment induced a root osmotic regulatory response, resulting in an increase in the proline contents and a decrease in malondialdehyde contents. Consequently, Homs-Schx promoted the growth of tomatoes and wheat in saline–alkali soil. This study provides new ideas for enhancing the rhizosphere colonization of plant growth-promoting bacteria, regulating the structure of microbial communities, and enhancing crop stress tolerance with the aid of green material strategies.

## 1. Introduction

Soil salinization is one of the major environmental stresses that restrict agricultural production and sustainable land use worldwide [1,2]. High salinity and high pH environments not only directly damage plant root functions by triggering osmotic stress, ionic toxicity and nutrient imbalance, but also disrupt the microbial community structure of the rhizosphere and inhibit soil enzyme activities, thereby severely restricting crop growth and yield [3,4,5,6]. Against the backdrop of increasingly tight global arable land resources and the continuous increase in food demand, the improvement and utilization of saline–alkali land have become important issues for ensuring food security and promoting sustainable agricultural development [7,8].

At present, there are three main techniques for improving the quality of saline–alkali land: physical improvement, chemical improvement and biological improvement. Although physical salt washing and the application of chemical improvers have achieved certain results, they still have limitations such as high cost, unstable effect, ease in causing secondary pollution, damage to soil ecology, imbalance of microbial communities and loss of organic matter [9,10,11]. Therefore, developing efficient, environmentally friendly and sustainable biological improvement strategies has become the current research focus in the field of saline–alkali land restoration. In recent years, plant rhizosphere growth-promoting bacteria have shown great potential in enhancing the salt–alkali tolerance of crops [12,13]. Among them, *Halomonas* (Homs), as a typical type of halophilic or salt-tolerant bacteria, can directly or indirectly alleviate the damage of saline–alkali stress to plants through multiple mechanisms such as producing osmotic protective substances, secreting plant hormones, and activating soil nutrients [14,15,16,17]. However, exogenous plant-promoting bacteria often have the problems such as low colonization efficiency and unstable survival in complex soil environments. These problems lead to the inability of their functional components to effectively accumulate around the root system and exert their effect for a long time [18,19,20].

To address the aforementioned bottlenecks, materials science offers new ideas for microbial delivery. Advanced materials such as metal–organic frameworks and hydrogels can achieve controllable release and targeted delivery by loading functional molecules or microorganisms [21,22]. For instance, the composite hydrogel constructed by sodium alginate and chitosan can not only improve soil structure and water retention, but also serve as a microbial carrier to enhance their survival and colonization capabilities. For instance, composite hydrogels composed of sodium alginate and chitosan have been widely applied in multiple fields such as biomedicine and pharmaceuticals, due to their porous, expandable, soft properties, as well as their ability to respond to various stimuli and biodegradability [23,24]. In recent years, the application prospects of polysaccharide hydrogel carrier materials in the field of agricultural chemicals have been very broad. They can not only improve soil structure and enhance its water retention capacity, but also serve as carriers for microorganisms, helping them survive better and colonize in the soil [25,26].

**Scheme 1:**
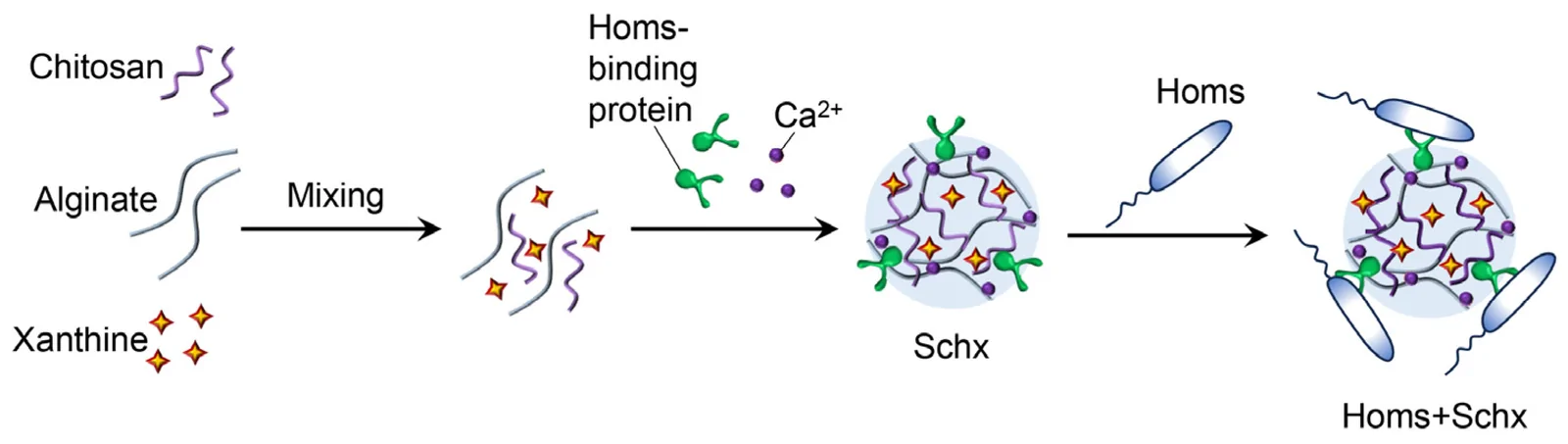
A scheme to illustrate the preparation of the *Halomonas* (Homs) and sodium alginate–chitosan–xanthine (Schx) assembly (Homs + Schx).

Based on this, the study designed and synthesized the smart hydrogel Schx, which is formed by mixing sodium alginate (S), chitosan (c), the artificial Homs-binding protein (h) and the plant stress-resistant molecule xanthine (x). This hydrogel not only has excellent soil-improving properties, but also promotes the recruitment of Homs cells on its surface, thereby forming a stable Homs-Schx assembly (Scheme 1). Through pot experiments, we systematically compared the effect of Homs alone, Schx alone, and the Homs-Schx assembly on crop growth, physicochemical properties of rhizosphere soil, microbial root colonization and community structures in saline–alkali soil. The results indicate that the Homs-Schx assembly promoted the colonization of Homs in the crop rhizosphere soil, thereby effectively regulating the rhizosphere microenvironment, alleviating saline–alkali stress, and ultimately promoting crop growth. This study aims to clarify the synergistic mechanism of “bacterium-hydrogel” assemblies in the improvement of saline–alkali soil, providing a theoretical basis and practical solution for the development of new biomaterial remediation technologies. This system is expected to offer new methods for increasing crop yields and improving saline–alkali soil in protected agriculture.

## 2. Results

### 2.1. Characterization of the Hydrogels and the Homs + Schx Assembly

**Figure 1:**
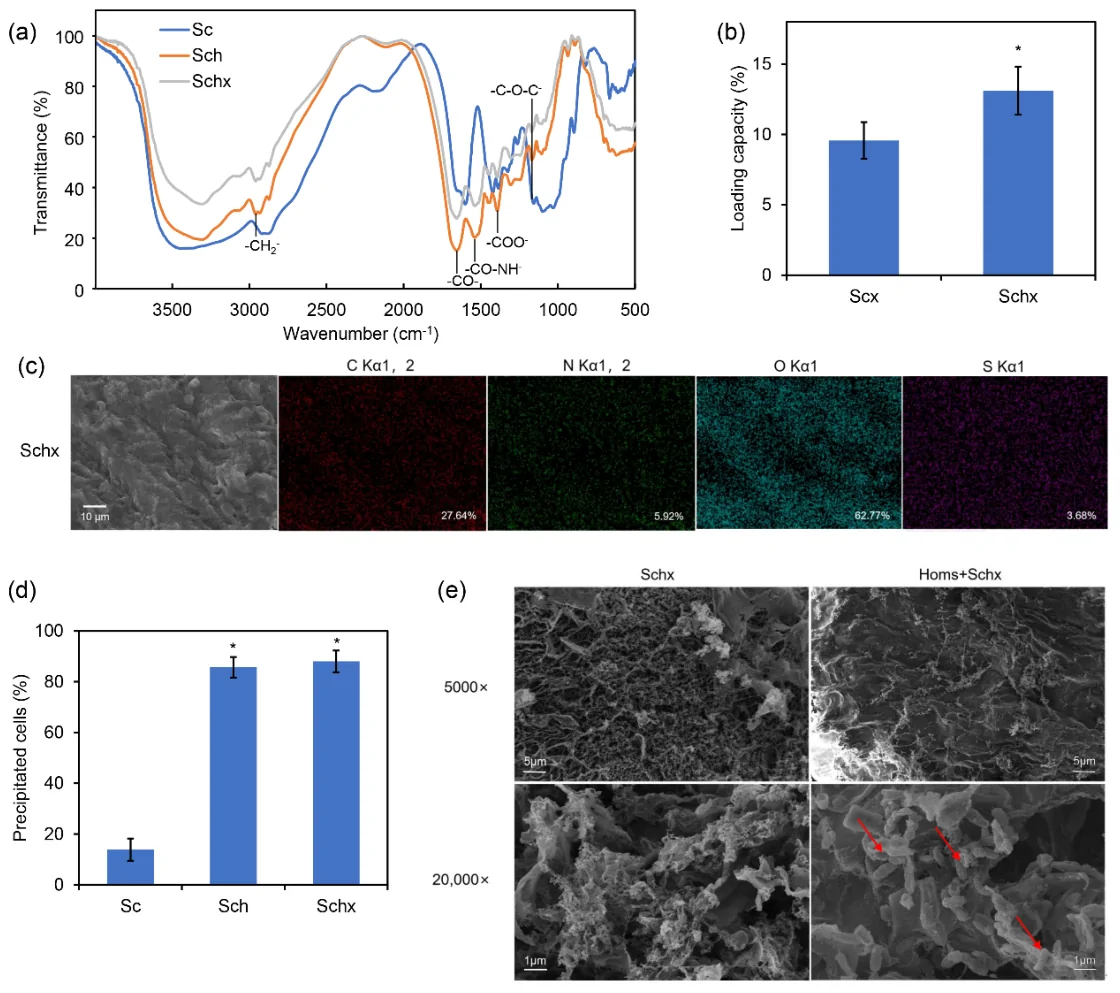
Characterization of Schx and Homs + Schx. (**a**) FT−IR spectra of Sc, Sch and Schx. (**b**) Xanthine−loading capacity of Scx and Schx. The asterisk (*) indicates significant difference between Sc and Sch or Schx (*p* < 0.05). (**c**) EDS mapping of Schx. (**d**) Precipitation of Homs cells induced by Sc, Sch and Schx. The asterisks (*) indicate significant difference between Sc and Sch or Schx (*p* < 0.05). (**e**) SEM images of Schx and Homs + Schx. The red arrows indicate the distribution of Homs cells.

To enhance the self-assembly of Homs cells and their colonization ability in the rhizosphere environment, we synthesized the Homs-binding protein-exposed sodium alginate–chitosan–xanthine hydrogel (Schx) to assist in Homs assembly. Firstly, sodium alginate, chitosan and xanthine were mixed to generate the gel precursor solution. The solution was then added drop by drop to the solution containing both Ca^2+^ and Homs-binding protein, forming the Schx hydrogel (Scheme 1). The morphologies of Schx alone and Homs + Schx were observed using scanning electron microscopy. Schx alone has a distinct porous structure. Homs + Schx has a large number of Homs cells attached to the porous structure. This indicates that Schx can promote the self-assembly of Homs. FT-IR analysis showed that the three tested hydrogel samples, i.e., the Sc sample not containing both Homs-binding protein and xanthine, the Sch sample not containing xanthine, and the Schx sample, had absorption peaks of -CH_2_-, -COO- and -C-O-C- (Figure 1a), indicating the existence of alginate and chitosan. Specially, the Sch and Schx samples had peaks of -CO-NH- and -CO- (Figure 1a), confirming the successful inclusion of Homs-binding protein. Loading assays further revealed that the protein-included Schx had a higher xanthine-loading capacity than the control Scx not containing the protein (13.1% versus 9.5%, Figure 1b). EDS mapping images confirmed the existence of C, N, O, and S in the Schx hydrogel (Figure 1c).

Given the presence of the artificial protein, the Homs cells may be recruited to the Schx hydrogel. To evaluate the ability of the hydrogels to bind Homs cells, the Homs-precipitating experiment was then performed in PBS. After 5 min of co-incubation, the Sc hydrogel only induced the precipitation of 15% Homs cells, while the protein-included hydrogels, i.e., Sch and Schx, induced the precipitation of >85% cells (Figure 1d). This indicated that the Homs-binding protein strongly bound the Homs cells to the hydrogel surface for co-precipitation. SEM observation further confirmed that the Homs + Schx had abundant Homs cells adhering to the hydrogel surface (Figure 1e). These results revealed the strong Homs-binding capacity of the Schx hydrogel.

### 2.2. The Schx Hydrogel Enhances Root Binding of Homs

**Figure 2:**
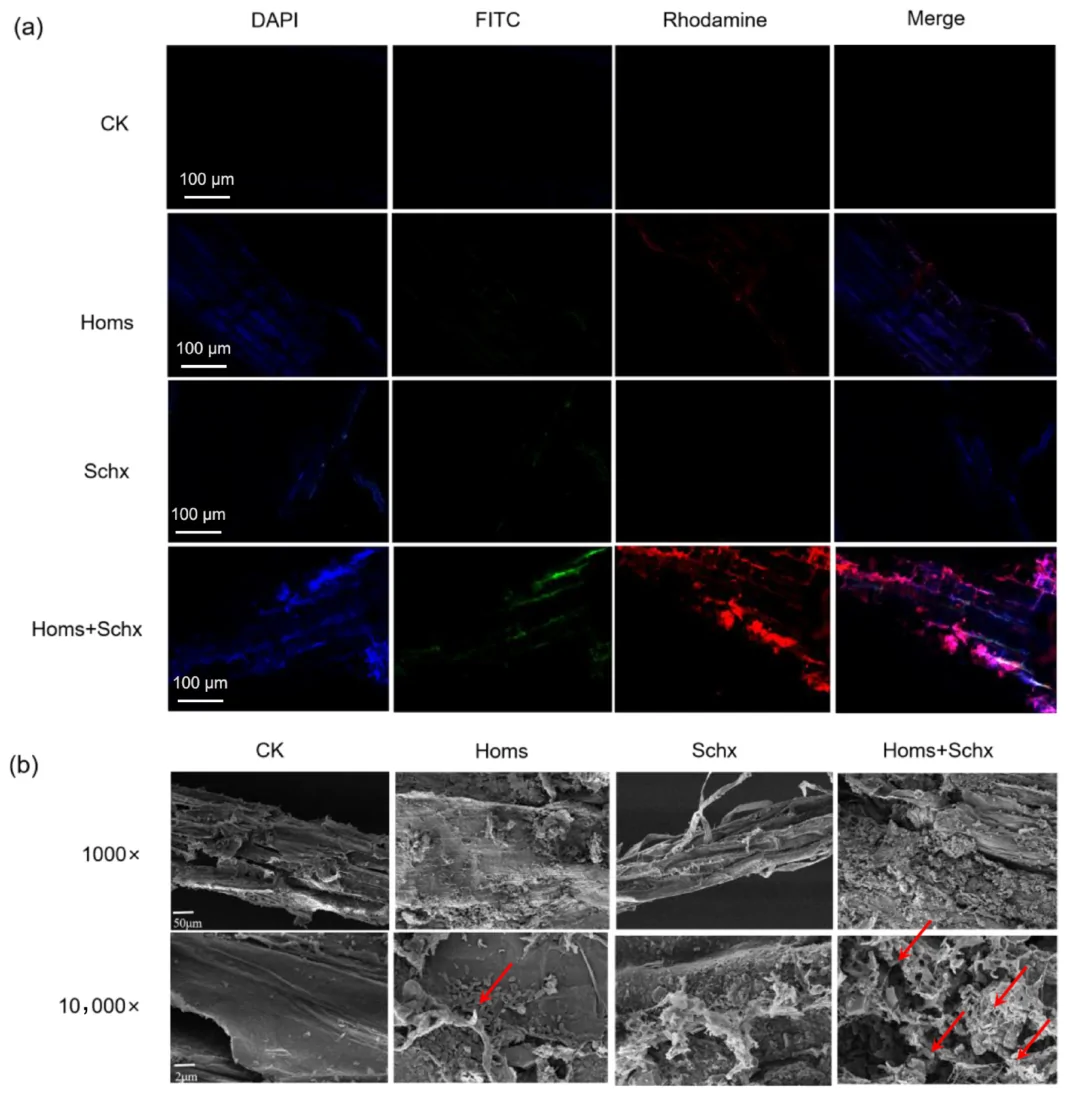
The effect of Homs, Schx or Homs + Schx on bacterial colonization in plant roots. (**a**) Confocal laser scanning microscopy images. DAPI (blue) stains the nuclei of Homs cells, FITC (green) indicates FITC-labeled Schx, and Rhodamine (red) corresponds to the Rhodamine-labeled Homs antibodies. (**b**) Scanning electron microscopy (SEM) images of the treated wheat roots. The red arrows indicate the Homs cells adhering to the root surface.

After 12 h co-culture of Homs with Schx and plant roots, confocal microscopy showed that the CK, Homs and Schx groups had no obvious fluorescence signals on the root surface, indicating that the colonization rate of Homs and Schx on plant roots was low when they were treated alone. In contrast, the Homs + Schx group had strong green and red fluorescence signals on the root surface, indicating that Homs and Schx closely interacted with each other, and Schx promoted the colonization of Homs on plant roots. (Figure 2a). The results of the SEM images are consistent with the above conclusion, which shows that the Homs + Schx group had much higher levels of Homs on the root surface (Figure 2b).

To further confirm the positive effect of Schx on Homs colonization at the plant roots, the wheat plants were cultured in sterilized soil containing 0.6% NaCl, and then Homs, Schx or Homs + Schx were added to the rhizosphere soil, followed by further cultivation for 30 days for Homs quantification. Colony-forming unit assays showed that the Homs + Schx group had much higher levels of Homs cells in the rhizosphere soil than the Homs group (8.3 × 10^6^ cells/g soil versus 3.5 × 10^6^ cells/g soil, Figure S1). Meanwhile, we determined the growth curves of Homs cells at a broad range of NaCl concentrations (0–20%, *w*/*v*). The results showed that the strain could grow at NaCl concentrations from 0 to 10% (Figure S2). Hence, Homs is not an obligate halophile but rather a halotolerant bacterium capable of growing under environments with a wide range of salt concentrations, indicating its potential for growth in the rhizosphere at salt concentrations. Together, these results revealed that Homs may survive in rhizosphere environments with different salt concentrations, and the Schx hydrogel strongly promoted Homs colonization in the rhizosphere soil.

### 2.3. Homs + Schx Assembly Alters the Composition of the Rhizosphere Microbiota

**Figure 3:**
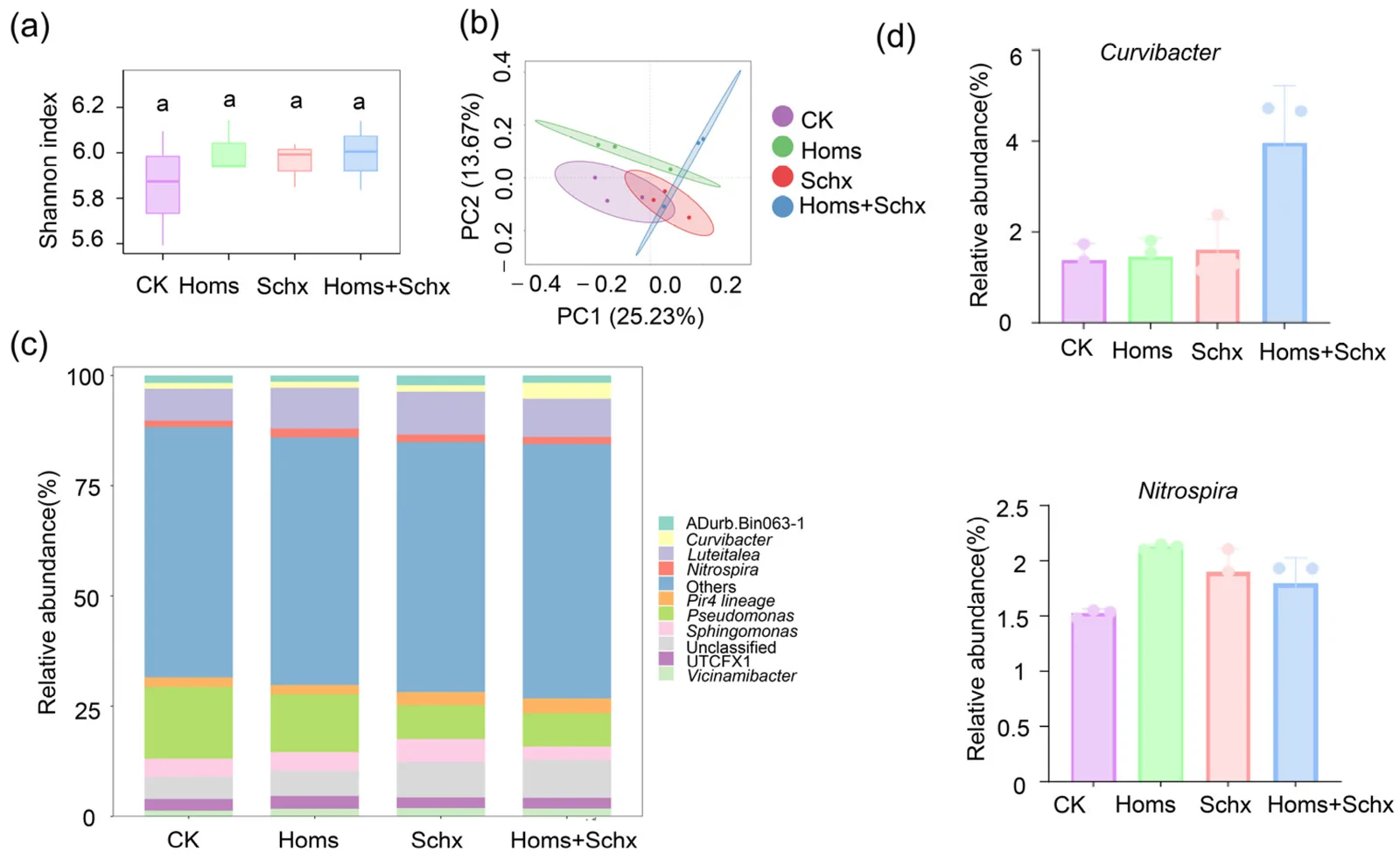
Microbiome analysis of the wheat rhizosphere soils by 16S rDNA sequencing in the four experimental treatments, i.e., the CK (untreated), the Homs, the Schx, and the Homs + Schx (combined treatment). (**a**) Shannon index of the four groups. Different lowercase letters indicate statistically significant differences among treatments at *p* < 0.05 (one-way ANOVA followed by Tukey’s HSD test). (**b**) Principal coordinate analysis (PCoA) of the microbiome composition in the four groups based on weighted UniFrac distances. PERMANOVA (999 permutations) confirmed significant differences among groups (*p* < 0.05). (**c**) Relative abundance of the bacterial groups between the treatments at the genus level. (**d**) The relative abundance of bacterial genera with significant differences under different treatments. Different lowercase letters indicate statistically significant differences among treatments at *p* < 0.05 (one-way ANOVA followed by Tukey’s HSD test).

To evaluate the potential of Homs and Schx in improving saline–alkali soil, we established four treatments for use during wheat cultivation: CK, Homs, Schx, and Homs + Schx. Sixty days later, rhizosphere soil samples were collected for 16S rDNA sequence analysis. The results indicated that the addition of Homs + Schx increased the Shannon index, suggesting that the addition of Homs and Schx could enhance the richness and stability of the microbial community (Figure 3a). Principal coordinate analysis (PCoA) confirmed significant differences in community composition among the groups (Figure 3b). Among them, the relative abundance of *Curvibacter* in the Homs + Schx group was significantly higher than that in the other groups, and the relative abundance of *Nitrospira* in the three treatment groups was significantly higher than that in the control group. *Nitrospira* has been reported to play a key role in nitrification and biofilm formation in various ecosystems [27,28]. Although the precise functional roles of these genera in alleviating saline–alkali stress in the rhizosphere remain to be fully elucidated, their enrichment in the Homs + Schx treatment suggests a potential link between the observed microbial community shifts and the improved soil conditions (Figure 3c,d).

### 2.4. The Homs + Schx Assembly Reduces the pH and Salt Content of the Rhizosphere Soil

**Figure 4:**
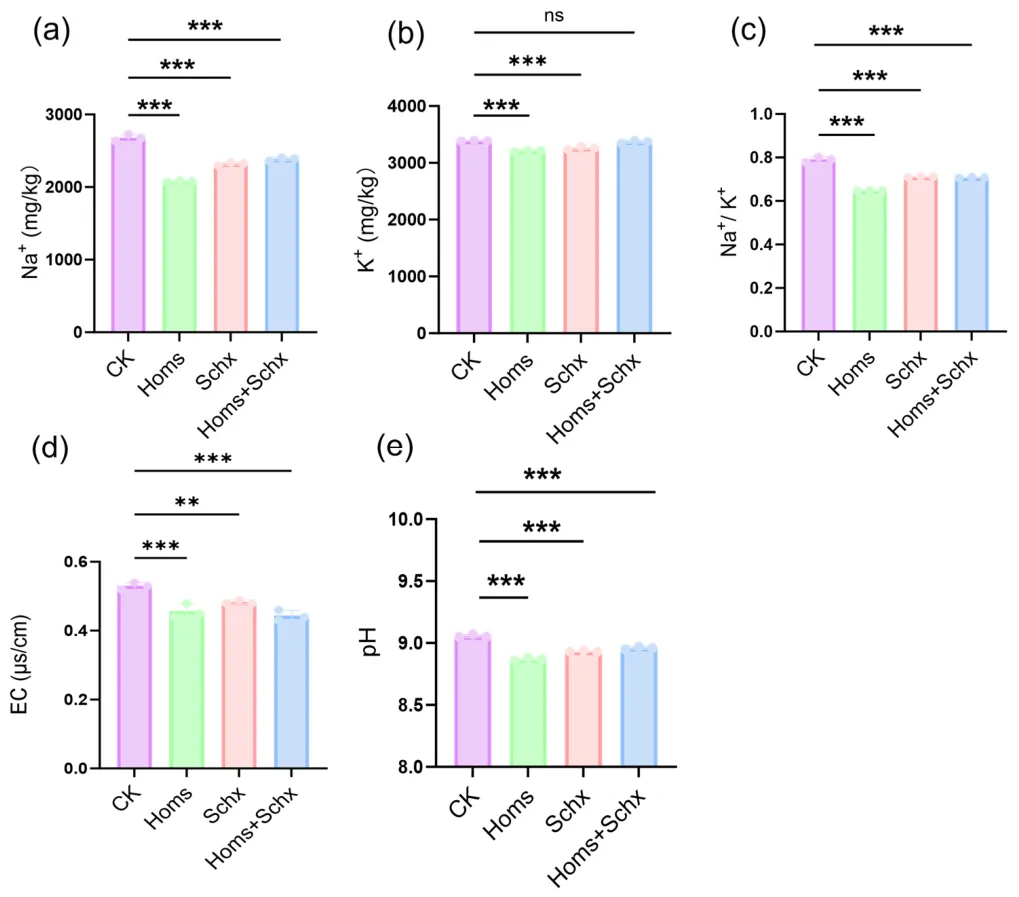
The effect of Homs, Schx and Homs + Schx on the ion content, pH and salt content in the wheat rhizosphere soil under the saline–alkali stress. (**a**) Na^+^ contents. (**b**) K^+^ contents. (**c**) Na^+^/K^+^ ratio. (**d**) Salt contents. (**e**) pH values. Data are presented as means ± SEM (*n* = 3). Statistical significance was determined by one-way ANOVA followed by Tukey’s HSD post hoc test. Asterisks indicate significant differences between the indicated groups (** *p* < 0.01, or *** *p* < 0.001, ns, not significant).

In saline–alkali soil environments, sodium ions (Na^+^) are the main factors causing ionic toxicity and osmotic stress in plants, while potassium ions (K^+^), as essential nutrients, play a crucial role in maintaining cellular osmotic balance and enzyme activity. Therefore, one of the important goals of an effective improvement strategy is to reduce the soil Na^+^/K^+^ ratio. In this study, compared with the control, the Homs-Schx group significantly reduced the Na^+^ contents (Figure 4a) and Na^+^/K^+^ ratio (Figure 4c) in the rhizosphere soil, while increasing the available K^+^ contents (Figure 4b), indicating that this assembly effectively regulated the ion imbalance state of saline–alkali soil. What is more worthy of attention is that Homs-Schx also significantly reduced the soil salt contents (Figure 4d) and pH values (Figure 4e), further demonstrating the comprehensive potential of this system in improving the overall saline–alkali stress environment.

### 2.5. Homs + Schx Optimizes Biochemical Indicators of Wheat and Enhances Stress Resistance

**Figure 5:**
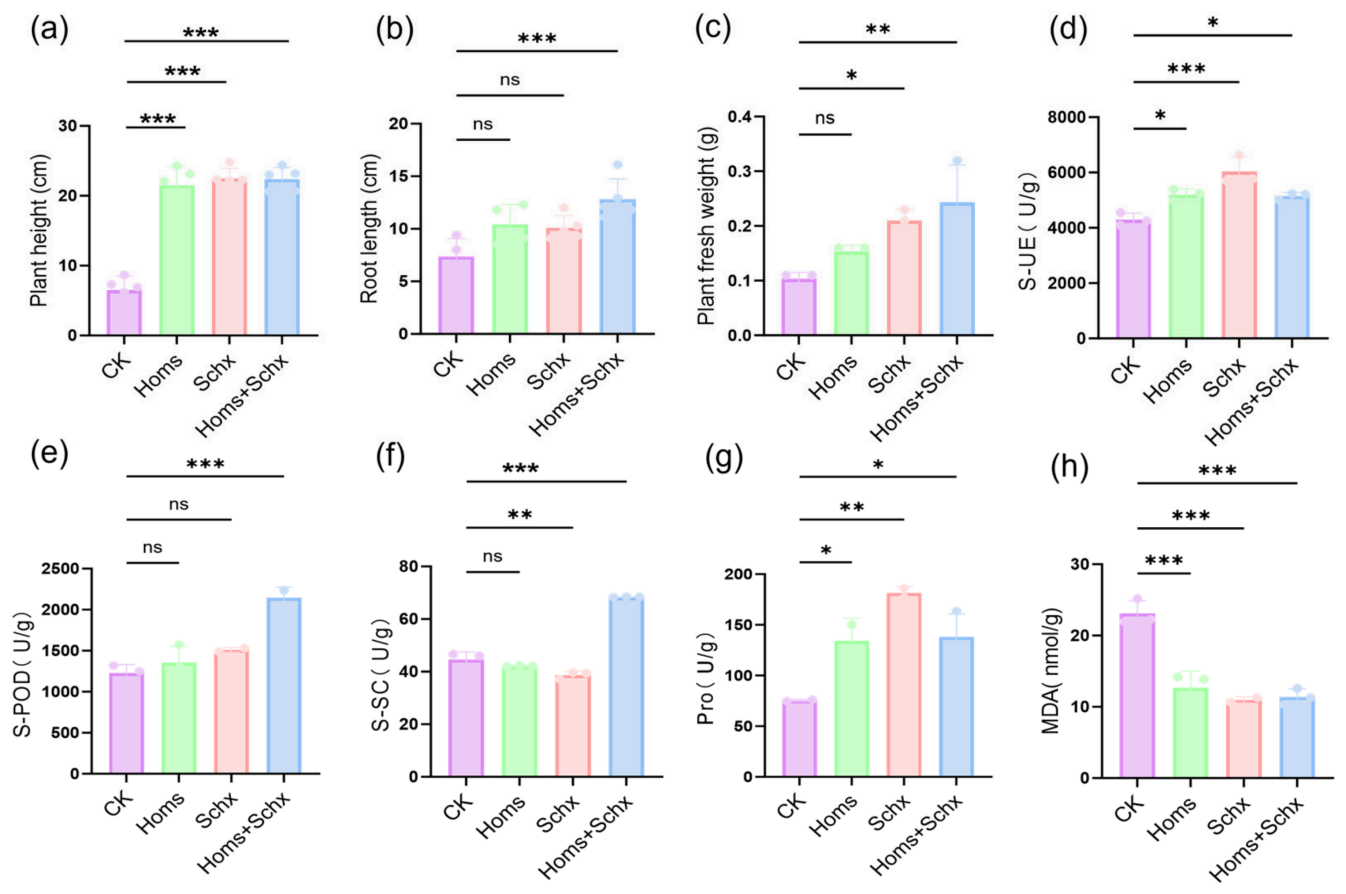
The effects of different treatments (CK, Homs, Schx, Homs + Schx) on wheat growth and root physiological indicators under saline–alkali stress. (**a**) Plant height. (**b**) Root length. (**c**) Plant fresh weight. (**d**) S-UE activity. (**e**) S-POD activity. (**f**) S-SC activity. (**g**) Root proline contents. (**h**) Root MDA contents. Data are presented as means ± SEM (*n* = 3). Statistical significance was determined by one-way ANOVA followed by Tukey’s HSD post hoc test. Asterisks indicate significant differences between the indicated groups (* *p* < 0.05, ** *p* < 0.01, or *** *p* < 0.001, ns, not significant).

The plant height, root length and fresh weight of wheat were measured after 30 days of cultivation in saline–alkali soil (Figure 5a–c). The results showed that after 30 days of saline–alkali stress, the plant height and root length in the Homs + Schx group were higher than those in the control group, and the combined treatment of Homs + Schx had the most significant effect. Then, the activities of rhizosphere enzymes in each group were detected. As shown in Figure 5d–f, the activities of soil urease (S-UE) in the three treatment groups were all higher than those in the control group. Even more encouragingly, the activities of soil peroxidase (S-POD) and soil sucrase (S-SC) in the Homs + Schx group were both higher than those in the other groups (Figure 5e–g). This indicates that Homs + Schx enhanced the soil enzyme activity under saline–alkali stress.

To assess the stress response of plants, the levels of proline (Pro) and malondialdehyde (MDA) in root tissues were measured. Proline is a crucial non-enzymatic osmotic regulator in the antioxidant mechanism and plays a significant role in alleviating salt stress. As shown in Figure 5g, the proline content in the treatment group was significantly higher than that in the control group. The results of malondialdehyde assays further indicated that the levels of malondialdehyde in the Homs + Schx group was the lowest among the four groups (Figure 5h), suggesting that the degree of plant tissue oxidation in the Homs + Schx group was the lowest. The above phenomena indicate that Homs + Schx enhanced the stress resistance of plants and was beneficial to plant growth. Therefore, the co-assembly can not only promote the morphological growth of plants but also alleviate the impact of saline–alkali stress on plant metabolism. This dual function highlights the potential of this comprehensive strategy in improving the performance and stress resistance of degraded agricultural soil crops.

### 2.6. Homs + Schx Promotes Tomato Growth and Improves the Quality of Saline–Alkali Soil

**Figure 6:**
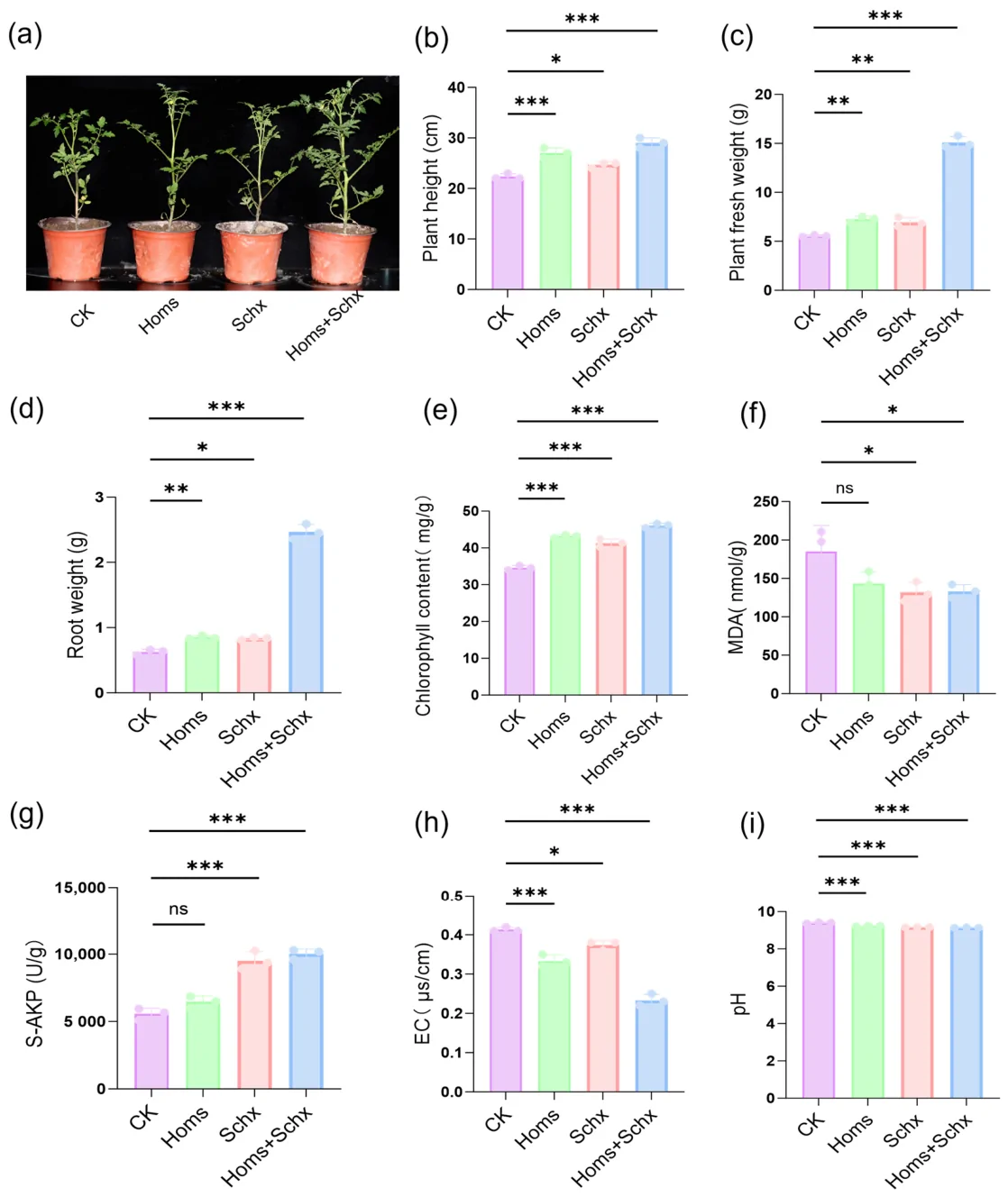
The effects of different treatments (CK, Homs, Schx, Homs + Schx) on tomato growth under saline–alkali stress. (**a**) Morphological comparison of tomato plants. (**b**) Plant height. (**c**) Plant fresh weight. (**d**) Root weight. (**e**) Chlorophyll content. (**f**) Root MDA contents. (**g**) S-AKP activity. (**h**) Salt content. (**i**) pH value. Data are presented as means ± SEM (*n* = 3). Statistical significance was determined by one-way ANOVA followed by Tukey’s HSD post hoc test. Asterisks indicate significant differences between the indicated groups (* *p* < 0.05, ** *p* < 0.01, or *** *p* < 0.001, ns, not significant).

After the tomato plants had been cultivated for the 30 days, it was observed that there was significant difference in the plant heights and weights among different groups. Among them, the Homs + Schx group exhibited the highest plant height and weight (Figure 6a–c). Meanwhile, the two control groups, i.e., the Hbp-free group Homs + Scx and the xanthine-free group Homs + Sch, had much lower plant heights than the Homs + Schx group (Figure S2), indicating the important role of Hbp and xanthine in the hydrogel. Furthermore, the root system of the Homs + Schx group was particularly developed, along with the root weight (Figure 6d). Subsequently, the chlorophyll content of the leaves was detected (Figure 6e). The results showed that the chlorophyll content of each treatment group was significantly higher than that of the control group. Meanwhile, the determination results of malondialdehyde (MDA) contents in the plant roots indicated that this treatment significantly reduced the MDA contents in the roots (Figure 6f), suggesting that Homs + Schx effectively alleviated the degree of oxidative damage to the cell membrane. In addition, the results of soil alkaline phosphatase assays showed that the enzyme activity in all three treatment groups was higher than that in the control group (Figure 6g), among which the Homs + Schx group had the most significant effect. Similarly, the Homs + Schx treatment significantly reduced the salt content and pH value of saline–alkali soil (Figure 6h,i).

### 2.7. Homs + Schx Increases the Abundance of Functional Microorganisms in Tomato Rhizosphere Soil

**Figure 7:**
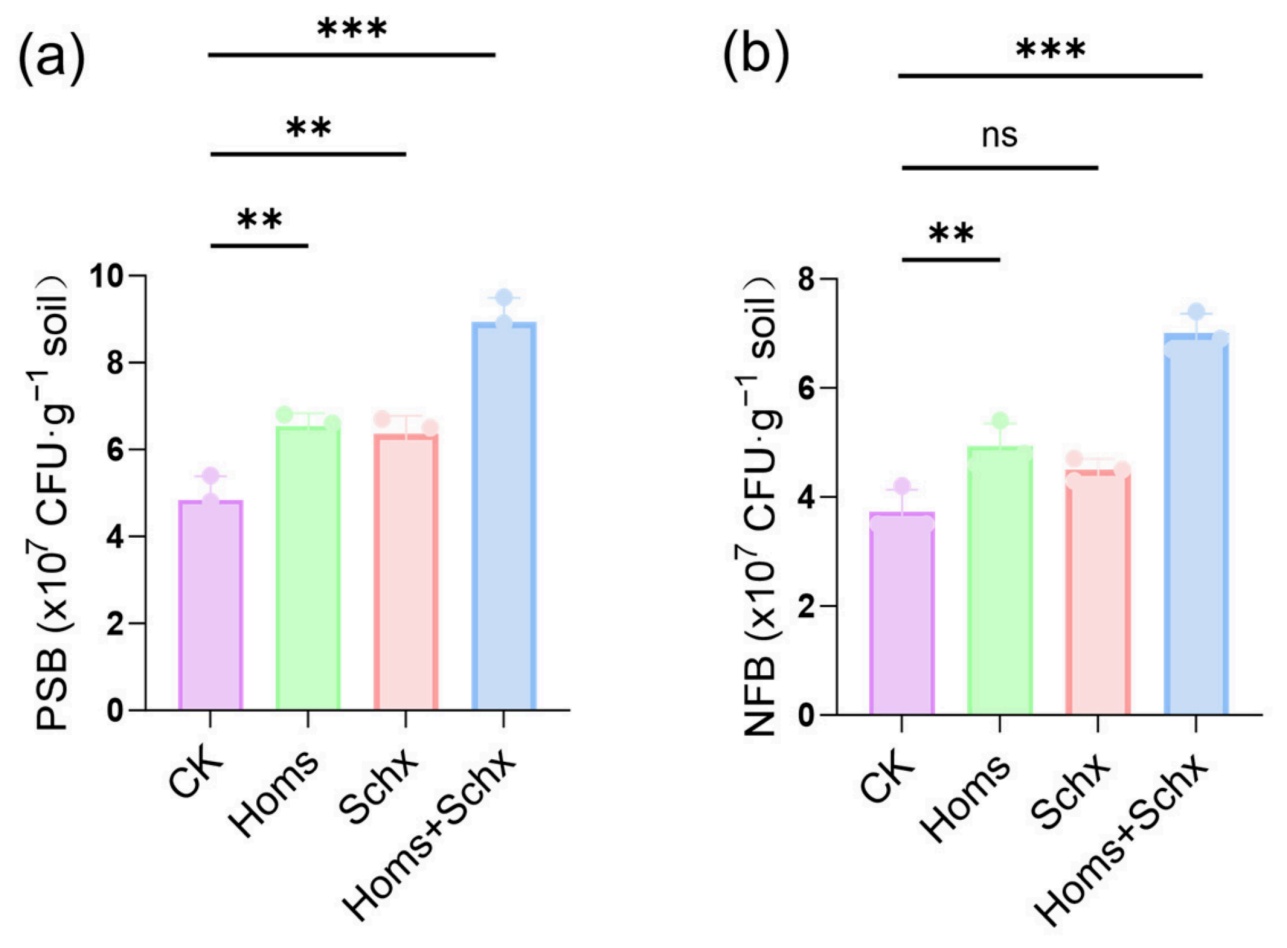
The abundance of functional microorganisms in the rhizosphere soil of tomato under different treatments (CK, Homs, Schx, Homs + Schx). (**a**) PSB abundance. (**b**) NFB abundance. Data are presented as means ± SEM (*n* = 3). Statistical significance was determined by one-way ANOVA followed by Tukey’s HSD post hoc test. Asterisks indicate significant differences between the indicated groups (** *p* < 0.01, or *** *p* < 0.001, ns, not significant).

To assess the abundance of functional microorganisms in the rhizosphere soil of tomato, we employed the colony-forming unit (CFU) assay to analyze the quantities of phosphorus-solubilizing bacteria (PSB) and nitrogen-fixing bacteria (NFB) under different treatments. Phosphorus-solubilizing bacteria (PSB) can convert the ineffective phosphorus in the soil that plants cannot directly utilize into absorbable effective phosphorus, thereby promoting the development of plant roots, flowering and fruiting. Nitrogen-fixing bacteria (NFB) can reduce nitrogen (N_2_) in the atmosphere to ammonia (NH_3_) or ammonium salts (NH_4_^+^), providing plants with nitrogen that can be directly utilized. Both can reduce the application of chemical fertilizers, improve soil structure, and help increase the yield and quality of crops [29,30,31,32]. To assess the abundance of functional microorganisms in the rhizosphere soil, we employed the colony-forming unit (CFU) assay to analyze the quantities of phosphorus-solubilizing bacteria (PSB) and nitrogen-fixing bacteria (NFB) under different treatments. As shown in Figure 7, the Homs + Schx group had much higher amounts of both PSB and NFB than the other three groups. For example, while the CK, Homs, and Schx groups had PSB amounts < 7 × 10^7^ CFU/g soil, the Homs + Schx group had PBS amounts of 9 × 10^7^ CFU/g soil (Figure 7a). These results indicate that the assembly is beneficial for PSB and NFB colonization in the rhizosphere region.

## 3. Discussion

Soil salinization is a severe challenge that restricts global agricultural production and sustainable development. Although traditional physical and chemical improvement methods have certain effects, they are often accompanied by problems such as high cost, significant environmental impact or poor sustainability. In recent years, the utilization of beneficial microorganisms (such as plant rhizosphere growth-promoting bacteria) to improve saline–alkali land has become an environmentally friendly and promising strategy. However, exogenous microorganisms have low colonization efficiency and their functions are difficult to sustain in complex soil environments, which seriously limits their practical application effects [33,34]. Sodium alginate is a by-product extracted from brown algae such as kelp or Sargassum, mainly containing two components: β-D-manuronic acid and α-L-guluronic acid. Sodium alginate forms a linear polymer through the connection of 1 to 4 glycosidic bonds [35]. This unique molecular structure enables them to cross-link with each other, thereby generating hydrogels. Hydrogels are three-dimensional network structures composed of hydrophilic polymers immersed in a large number of water molecules which possess dual solid and liquid characteristics. They have the ability to expand without dissolving [36]. This material is widely used in fields such as tissue engineering and biomedicine [37,38,39].

In this study, a microbe-material functional assembly (Homs-Schx) based on a sodium alginate–chitosan–xanthine composite hydrogel (Schx) was constructed to address the problem of rhizosphere colonization of the salt-tolerant strain *Halomonas*. This assembly significantly enhances the resistance of the crop to saline–alkali stress through material-mediated targeted delivery and the synergistic regulation of rhizosphere microecology, providing a new material-microbial synergistic strategy for the bioremediation of saline–alkali land.

The results of this study indicate that the Schx material can effectively load and protect Homs cells, and form a stable “material—mycelium—root system” composite structure on the root surface of wheat. Confocal and scanning electron microscopy observations showed that the Homs-Schx treatment group formed a dense biofilm-like structure on the root surface, while the colonization effect was limited when Homs or Schx was applied alone. We hypothesize that the enhanced colonization of Homs cells may be attributable to the porous structure of Schx, its surface charge characteristics, and its potential affinity with root exudates, which together facilitate bacterial adhesion and microhabitat formation. As a natural polysaccharide hydrogel, Schx may not only provide physical protection and a microhabitat for the bacteria, but also enhance adhesion between the bacteria and the root surface through mechanisms such as hydrogen bonding and electrostatic interactions. This is consistent with recent studies that have utilized carriers such as ZIF-8 and biochar to enhance the colonization efficiency of PGPR [20]. However, direct evidence for these proposed mechanisms—such as Zeta potential measurements, bacterial adhesion assays, and root exudate composition analysis—was not obtained in the current study and needs further investigation.

The rhizosphere microbial community is a key regulator of plants in response to saline–alkali stress. In this study, Homs + Schx treatment significantly altered the rhizosphere bacterial community structure and increased the relative abundance of bacterial genera related to the nitrogen cycle (e.g., *Nitrospira*) and biofilm formation (e.g., *Curvibacter*). While the enrichment of these taxa correlated with improved soil parameters, we acknowledge that the current 16S rRNA sequencing data do not establish causal relationships. Traditionally, studies on *Nitrospira* have predominantly focused on wastewater treatment and estuarine biofilms [40,41,42]. Furthermore, recent evidence indicates that *Nitrospira* can dominate nitrification in biofilm niches [27] and exhibit alkali-resistance mechanisms under high-pH conditions relevant to saline–alkali soils [28]. These findings suggest a potential role of *Nitrospira* in saline–alkali rhizosphere environments. Nevertheless, the inference that increased *Nitrospira* abundance directly alleviates saline–alkali stress needs to be further investigated.

To move beyond a general description of treatment effects and gain deeper mechanistic insights, we analyzed the correlative relationships among the key parameters measured in this study. Firstly, the reduction in soil pH and salt content (Figure 4d,e and Figure 6h,i) was closely associated with decreased Na^+^ content and Na^+^/K^+^ ratio (Figure 4a–c), suggesting that ion regulation is a primary pathway through which the Homs + Schx assembly improves soil chemistry. This ion balance restoration likely reduces the ionic toxicity and osmotic stress experienced by plant roots, as reflected by the significantly lower malondialdehyde (MDA) contents in the Homs + Schx group (Figure 5h and Figure 6f). Secondly, soil enzymes are widely recognized as sensitive indicators of soil fertility, nutrient transformation, and ecosystem function under environmental stress. For instance, soil urease (S-UE) activity reflects soil nitrogen mineralization potential, while soil sucrase (S-SC) activity governs the hydrolysis of sucrose into glucose and fructose, providing carbon sources for rhizosphere microorganisms. Moreover, soil peroxidase (S-POD) contributes to the detoxification of reactive oxygen species in the rhizosphere, and alkaline phosphatase (S-AKP) mediates organic phosphorus mineralization [43,44,45,46]. In our study, the Homs + Schx treatment significantly elevated the activities of S-UE, S-POD, S-SC (Figure 5d–f), and S-AKP (Figure 6g). Notably, the increased abundance of functional microorganisms—particularly phosphorus-solubilizing bacteria (PSB) and nitrogen-fixing bacteria (NFB) (Figure 7)—showed a positive correlation with these enhanced enzyme activities, suggesting that the enriched functional microbes contribute to nutrient cycling (nitrogen, carbon, and phosphorus) and redox metabolism in the rhizosphere, thereby supporting plant growth under saline–alkali stress. Thirdly, the increased chlorophyll content in tomato leaves (Figure 6e) correlated positively with plant height, shoot length, fresh weight, and root weight (Figure 6b–d), indicating that improved photosynthetic capacity directly contributed to biomass accumulation. This is consistent with the well-established role of chlorophyll as an indicator of plant vigor and stress tolerance [47,48]. Fourth, the enhanced root length and biomass observed in both wheat and tomato (Figure 5a–c and Figure 6b–d) were inversely correlated with root MDA contents, suggesting that reduced oxidative damage facilitates root development, which in turn improves water and nutrient uptake. Soil pH, which was significantly reduced by the Homs + Schx treatment (Figure 4e and Figure 6i), showed a positive correlation with salt content, further confirming that the pH reduction is accompanied by amelioration of soil salinity.

**Figure 8:**
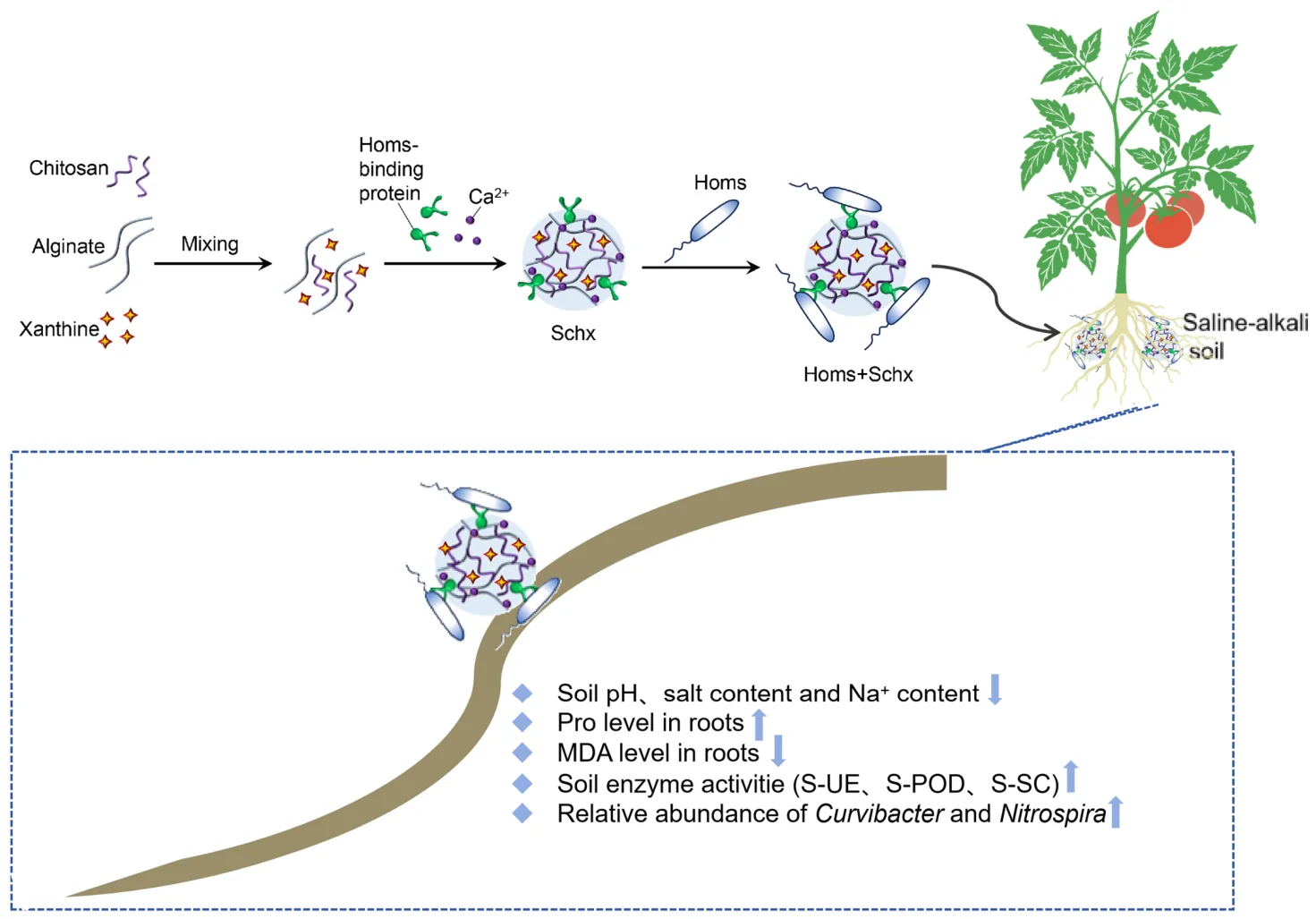
A scheme illustrating the preparation process of Homs-Schx and the way it colonizes in the rhizosphere and regulates the rhizosphere microbial community, thereby reducing soil salinity and alkalinity, increasing soil enzyme activity and enhancing the stress resistance of plants.

Collectively, these correlative patterns support a sequential model in which the Homs + Schx treatment initiates a cascade of beneficial effects. The initial improvement in soil chemical properties, including reduced pH, decreased salt content, and a lower Na^+^/K^+^ ratio, is followed by the enrichment of functional microorganisms (PSB and NFB) and the enhancement of soil enzyme activities (S-UE, S-POD, S-SC, and S-AKP). These microbial and biochemical changes subsequently alleviate plant oxidative stress, as evidenced by decreased MDA levels and increased proline accumulation, which in turn promote photosynthetic capacity (indicated by elevated chlorophyll content) and root system development (increased root length and root weight). Ultimately, these coordinated improvements culminate in enhanced overall plant growth, as reflected by increased plant height, shoot length, and biomass. In summary, the Homs-Schx co-assembly not only supports rhizosphere colonization and synergistic microbial interactions, but also offers a promising strategy for improving soil quality and crop performance in saline–alkali environments (Figure 8).

Several mechanisms may contribute to the observed improvements in soil physicochemical properties and plant stress responses. Firstly, the Schx hydrogel and Homs cells may collaboratively adsorb or buffer excess salt ions in the rhizosphere, although quantitative adsorption isotherms and ion-exchange capacity measurements are needed to confirm this hypothesis. Secondly, Homs may secrete organic acids or extracellular polymeric substances that reduce localized pH and enhance nutrient solubilization. Thirdly, the improved rhizosphere microenvironment may promote nutrient and water uptake by plant roots, indirectly enhancing stress tolerance. These proposed mechanisms are speculative and require targeted experimentation—including Zeta potential analysis, adsorption isotherm studies, organic acid profiling, and functional metagenomics—to establish definitive cause-and-effect relationships.

Beyond these specific mechanistic hypotheses, several broader limitations of the present study should also be acknowledged. The mechanistic interpretations regarding material–bacteria–root interactions, ion regulation, and microbial functionality are primarily based on correlative evidence and literature-derived inferences, rather than direct experimental validation. While the observed phenotypic and physicochemical improvements are robust, the underlying molecular and biophysical mechanisms remain to be conclusively demonstrated. Future studies incorporating functional genomics, metabolomics, and advanced material characterization techniques will be essential to fully unravel the synergistic mechanisms of the “bacterium–hydrogel” assembly in saline–alkali soil improvement. Moreover, it should be noted that while this study employed two distinct crop species—wheat (a monocot) and tomato (a dicot)—to evaluate the broad-spectrum applicability of the Homs + Schx assembly, the detailed mechanistic analyses (microbiome sequencing, soil enzyme profiling, and comprehensive physiological measurements) were primarily conducted on wheat, with tomato serving as a validation crop for growth-promoting and soil-improving effects. Although a positive effect was consistently observed in both species, the physiological and rhizosphere-structural differences between monocots and dicots [49] may influence the magnitude and specific pathways of the beneficial effect. Future studies comparing the rhizosphere microbial responses of different crop species to the Homs + Schx assembly would be valuable to further establish its general applicability in protected agriculture.

## 4. Materials and Methods

### 4.1. Materials

The *Halomonas* strain *Halomonas ventosae* CICC 24079 (Homs) was obtained from the China Center of Industrial Culture Collection (Beijing, China). This strain can enhance the growth ability of plants under saline–alkali conditions. Sodium alginate was purchased from Aladdin, Shanghai, China. Chitosan was purchased from Shanghai Yuanye Bio-Technology, China. Xanthine was purchased from HEOWNS, Tianjin, China. The strains and the plasmids used in this study are outlined in Table S1. The Homs-binding protein (Hbp, 62 kD, Table S2) was designed on the basis of the *Homo sapiens* monocyte differentiation antigen CD14 sequence, which has high affinity to lipopolysaccharides of Gram-negative bacteria [50]. The *HBP* gene was further designed by using the optimal codons of *Saccharomyces cerevisiae* and de novo synthesized by QYAOBIO, Ltd. (Wuhan, China). This obtained fragment was then cloned into the yeast expressing plasmid pESC-URA. The plasmid was then transformed into the *S. cerevisiae* InvSc1 strain with the lithium acetate method, obtaining the synthetic yeast strain ScHbp (Table S2). The strain was cultured in YPD medium at 30 °C for 24 h, and then broken by grinding with the aid of liquid nitrogen. The Hbp protein was purified by nickel columns. The Im liquid medium formula for culturing Homs is as follows: glucose 0.05% (*W*/*V*), tryptone 0.4%, K_2_HPO_4_ 0.05%, MgSO_4_ 0.01%, Yeast Extract 0.4%, NaCl 10%, salt pool water 20%.

### 4.2. Synthesis and Characterization of Schx

To synthesize the Schx hydrogel, 1 g sodium alginate, 0.5 g chitosan and 0.1 g xanthine were dissolved in 100 mL distilled water under magnetic stirring at 600 rpm for 5 min. The mixture was then inhaled into syringes and added drop by drop into the solution containing 1 g/L Homs-binding protein and 10 g/L calcium chloride under gentle stirring. The hydrogel beads were harvested by gauze filtering. The hydrogel was dehydrated using a freezing vacuum drier. The morphology and energy dispersive spectrometry (EDS) mapping of Schx was characterized by scanning electron microscopy (TESCAN MIRA LMS, Burno, Czech Republic). The functional groups were detected with a Fourier transform infrared (FT-IR) spectrometer (Thermo Scientific Nicolet iS20, Waltham, MA, USA).

### 4.3. Plant Cultivation

The two crop species, i.e., wheat and tomato, were used in this study to evaluate the broad-spectrum applicability of the Homs + Schx assembly. Wheat was used for the detailed mechanistic analyses, including rhizosphere microbiome sequencing (Section 2.3), soil physicochemical property measurements (Section 2.4), soil enzyme activity assays, and plant physiological parameter determinations (Section 2.5). Tomato was used to further validate the growth-promoting effects and soil quality improvement in a different crop species (Section 2.6). The abundance of functional microorganisms (PSB and NFB) in the rhizosphere soil of tomato plants was evaluated (Section 2.7). The wheat and tomato seedlings were planted in pots containing nutrient-rich soil. Each pot was watered with 150 mL of saline–alkali solution to artificially create saline–alkali stress. The saline–alkali solution contained 0.6% (*m*/*v*) sodium chloride, 0.2% sodium sulfate, and 0.2% NaHCO_3_, with the pH adjusted to 9.0. Four treatment groups were established, with three replicate pots per group, for each crop species: (1) Control group (CK), (2) Homs group (1 × 10^8^ Homs cells/pot), (3) Schx group (100 mg/pot of Schx hydrogel), and (4) Homs + Schx group (1 × 10^8^ Homs cells/pot combined with 100 mg/pot of Schx). The plants were cultivated at room temperature under natural sunlight for 30 days. At harvest, plant height, root length, fresh weight, and chlorophyll content (for tomato) were measured. Plant tissues (leaves, stems, and roots) and rhizosphere soil samples were collected separately for further analysis.

### 4.4. Interactions Among Homs, Schx and Plant Roots

Fresh roots were taken from wheat seedlings and rinsed with running water to remove soil particles adhering to the roots. Then, the clean roots were suspended in PBS buffer and Homs and Schx were added to the suspension either separately or in combination. The final concentrations of Homs and Schx were 1 × 10^7^ cells/mL and 100 µg/mL, respectively. Then, the suspension was incubated at 28 °C for 24 h, and samples were taken and washed twice with deionized water. The obtained clean roots were soaked in a 4% formalin solution for 12 h. Subsequently, the tissues were successively immersed in ethanol solutions of 30%, 50%, 70% and 90% concentrations, each for 1 h, to dehydrate the samples. Subsequently, the tissue samples were freeze-dried and prepared for SEM (SEM, TESCAN MIRA LMS, Brno-Kohoutovice, Czech Republic) observation.

### 4.5. Rhizosphere Microbiome Analysis

We collected rhizosphere soil samples of plants that had undergone different treatments (CK group, Homs group, Schx group, and Homs + Schx group). Subsequently, total rhizosphere microbial DNA was extracted from each sample using the TGuide S96 magnetic bead extraction kit. Then, the bacterial 16S V3+V4 primer pairs (338F, 5′-ACTCCTACGGGAGGCAGCA-3′; 806R, 5′-GGACTACHVGGGTWTCTAAT-3′) were used to construct the bacterial 16S rDNA library (for analyzing bacterial diversity). The constructed library was sequenced by a biotechnology company in China. Data from each group were analyzed and processed using Biocloud software (url: http://www.biocloud.net, accessed on 9 June 2025).

### 4.6. Biochemical Analysis

For soil enzyme assays, rhizosphere soil samples were collected from each plant by gently shaking off loosely adhering soil and carefully collecting the soil tightly attached to the root surface using a sterile brush. The collected soil samples were passed through a 30–50 mesh sieve to remove plant debris and stones. The sieved soil samples were air-dried at room temperature (25 ± 2 °C) to constant weight, and stored at 4 °C prior to analysis. All soil enzyme activities were determined using commercial assay kits (Solarbio, Beijing, China) following the manufacturer’s protocols. Absorbance was measured using a visible spectrophotometer at the specified wavelengths. Soil urease (S-UE) activity was assayed using a Soil Urease (S-UE) Activity Assay Kit (Solarbio, BC0120, Beijing, China) based on the indophenol blue colorimetric method. One unit of S-UE activity was defined as the amount of enzyme that produces 1 µg of NH_3_-N per gram of dry soil per day under the assay conditions. Soil peroxidase (S-POD) activity was assayed using a Soil Peroxidase (S-POD) Activity Assay Kit (Solarbio, BC0890, Beijing, China). The method is based on the oxidation of organic substances catalyzed by S-POD, producing quinones that have characteristic light absorption at 430 nm. One unit of S-POD activity was defined as the amount of enzyme that produces 1 mg of purpurogallin per gram of dry soil per day under the assay conditions. Soil sucrase (S-SC) activity was assayed using a Soil Sucrase (S-SC) Activity Assay Kit (Solarbio, BC0240, Beijing, China) based on the 3,5-dinitrosalicylic acid (DNS) colorimetric method. One unit of S-SC activity was defined as the amount of enzyme that produces 1 mg of reducing sugar per gram of dry soil per day at 37 °C under the assay conditions. Soil alkaline phosphatase (S-AKP/ALP) activity was assayed using a Soil Alkaline Phosphatase (S-AKP/ALP) Activity Assay Kit (Solarbio, BC0280, Beijing, China) based on the disodium phenyl phosphate colorimetric method. One unit of S-AKP activity was defined as the amount of enzyme that releases 1 nmol of phenol per gram of dry soil per day at 37 °C under the assay conditions.

For the determination of plant physiological parameters, root tissues were collected from each plant at the end of the cultivation period. The roots were carefully washed with distilled water to remove adhering soil particles, blotted dry with filter paper, and immediately frozen in liquid nitrogen. The frozen root samples were ground to a fine powder in a pre-chilled ceramic mortar with liquid nitrogen. The powdered tissues (approximately 0.1 g) were homogenized with 1 mL of ice-cold extraction buffer (provided in the kits) and then placed in a boiling water bath for 10 min. After cooling, the homogenate was centrifuged at 10,000× *g* for 10 min at room temperature, and the supernatants were collected for analysis. Proline content was determined using a Proline (Pro) Content Assay Kit (Solarbio, BC0290, Beijing, China) based on the acid ninhydrin colorimetric method. Proline content was calculated based on tissue fresh weight and expressed as µg proline per gram of fresh weight (FW). Malondialdehyde (MDA) content was measured using a Malondialdehyde (MDA) Content Assay Kit (Solarbio, BC6415, Beijing, China) based on the thiobarbituric acid (TBA) method (TBARS detection). The MDA content was calculated using the formula MDA (nmol/g FW) = 32.258 × ΔA/W, where ΔA = (A_532_ − A_600_) − (A_532_(blank) − A_600_(blank)), and W is the sample fresh weight (g). The chlorophyll content of leaves was determined directly with a chlorophyll meter. Results were expressed as nmol MDA per gram of fresh weight (FW). All assays were performed in triplicate for each biological replicate, and the results are expressed as means ± standard errors (SEM).

### 4.7. Cation Quantification of the Rhizosphere Soils

To detect soil ion contents, the samples underwent mechanical homogenization using Dounce tissue grinders (tight-clearance, 50 µm) prior to acid digestion in 30% (*v*/*v*) nitric acid at 95 ± 1 °C under reflux conditions for 30 min intervals. Digestates were processed through gravimetric dilution protocol (1:10 *v*/*v*) with ultrapure water before elemental quantification via high-resolution ICP-MS (Elan DRC-II, PerkinElmer Inc., Waltham, MA, USA) with method blank verification.

### 4.8. Microbiome Analysis

The raw 16S rDNA sequencing data were processed using the QIIME 2 pipeline (version 2023.5). Raw reads were demultiplexed, quality-filtered, and denoised using the DADA2 plugin to generate amplicon sequence variants (ASVs). Taxonomic assignment was performed using the SILVA 138.2 reference database with a 99% similarity threshold [51]. The Shannon index was calculated to estimate the richness and evenness of the microbial community within each sample. Differences in the Shannon index among treatment groups were assessed using one-way analysis of variance (ANOVA), followed by Tukey’s honestly significant difference (HSD) post hoc test for multiple comparisons. Principal coordinate analysis (PCoA) was performed based on weighted UniFrac distance matrices to visualize the dissimilarities in microbial community composition among samples. To statistically evaluate the significance of inter-group differences, permutational multivariate analysis of variance (PERMANOVA) with 999 permutations was applied using the vegan package in R (version 4.3.0). A *p*-value of <0.05 was considered to indicate significant differences in community structure among treatment groups. The relative abundance of bacterial taxa at the genus level was calculated for each sample. To identify taxa with significantly different relative abundances among the four treatment groups, one-way ANOVA followed by Tukey’s HSD post hoc test was performed. Taxa with an average relative abundance below 0.1% across all samples were excluded from the differential analysis to reduce noise from rare taxa. Statistical significance was set at *p* < 0.05.

### 4.9. Statistical Analysis

All experiments were performed with three independent biological replicates, and each replicate was measured in triplicate (technical replicates). Data are presented as means ± standard errors (SEM). For comparisons involving more than two groups, one-way analysis of variance (ANOVA) was applied, followed by Tukey’s honestly significant difference (HSD) post hoc test for multiple comparisons. For comparisons between two groups, Student’s *t*-test was used. For microbiome data, Shannon index comparisons and genus-level differential abundance analysis were performed using one-way ANOVA with Tukey’s HSD test, while community-level differences (PCoA) were evaluated using PERMANOVA (999 permutations) based on weighted UniFrac distances, as detailed in Section 4.8. All statistical tests were two-sided, and a *p*-value of <0.05 was considered statistically significant. All analyses were performed using SPSS software (version 22.0, IBM Corp., Armonk, NY, USA) and R software (R Core Team, Vienna, Austria, version 4.3.0) with the vegan package.

## 5. Conclusions

In conclusion, this study successfully constructed the functional assembly Homs-Schx, which is composed of the salt-tolerant strain Homs and the natural polysaccharide hydrogel Schx. The assembly effectively promoted the directional colonization of Homs in the crop rhizosphere. By regulating the microbial community structure in the rhizosphere, increasing soil enzyme activity, improving ion balance and enhancing plant physiological resistance, the assembly alleviated the inhibitory effect of saline–alkali stress on crop growth, and improved plant biomass and stress adaptability. In this study, a new strategy for saline–alkali soil bioremediation based on material-microbial cooperation was developed which provided not only an effective technical method for improving crop salt tolerance, but also a theoretical reference for the design of new intelligent agricultural microbial preparations. The assembly system has the advantages of simple preparation, environmental friendliness and good application potential. In the future, through field experiments and carrier optimization, this technology is expected to become an important support for the improvement of agricultural green yield and the sustainable utilization of saline–alkali land.

## Data Availability

The original contributions presented in the study are included in the article/Supplementary Material. The raw data of microbiome analysis are available from the corresponding author on reasonable request.

## Supplementary Materials

The following supporting information can be downloaded at https://www.mdpi.com/article/10.3390/molecules31183215/s1.
