# Supplementary material for: The Assembly of Alginate–Chitosan–Xanthine Hydrogel and Halomonas Improves Soil Quality of Protected Crop Growth Under Saline–Alkali Stress
Source: Molecules. 2026 Sep 11;31(18):3215. doi: 10.3390/molecules31183215 (PMC13609178; doi:10.3390/molecules31183215)
Supplement: Supplementary file 1 [file molecules-31-03215-s001.zip › molecules-4443208-supplementary.pdf]

Supporting Information

Table S1 The strains and plasmids used in this study

Name	Genotype	Source
InvSc1	*his3-/-, leu2-/-, trp1-/-, ura3-/-*	Invitrogen, USA
ScHbp	*his3-/-, leu2-/-, trp1-/-, ura3-/-, HBP, URA3*	This study
pESC-URA	Amp^R, *URA3*	QYAOBIO, China
pESC-*HBP*	Amp^R, *URA3, HBP*	This study

Table S2 Sequence of the artificial protein Hbp and its engineering gene *HBP*

Name	Sequence	
Hbp	1	PEPCELDDED FRCVCNFSEP QPDWSEAFQC VSAVEVEIHA GGLNLEPFLK RVDADADPRQ
	61	YADTVKALRV RRLTVGAAQV PAQLLVGALR VLAYSRLKEL TLEDLKITGT MPPLPLEATG
	121	LALSSLRLRN VSWATGRSWL AELQQWLKPG LKVLSIAQAH SPAFSCEQVR AFPALTSLDL
	181	SDNPGLGERG LMAALCPHKF PAIQNLALRN TGMETPTGVC AALAAAGVQP HSLDLSHNSL
	241	RATVNPSAPR CMWSSALNSL NLSFAGLEQV PKGLPAKLRV LDLSCNRLNR APQPDELPEV
	301	DNLTLDGNPF LVPGTAGGGG GGVSKGEELF TGVVPILVEL DGDVNGHKFS VSGEGEGDAT
	361	YGKLTLKFIC TTGKLPVPWP TLVTTLTYGV QCFSRYPDHM KQHDFFKSAM PEGYVQERTI
	421	FFKDDGNYKT RAEVKFEGDT LVNRIELKGI DFKEDGNILG HKLEYNYNSH NVYIMADKQK
	481	NGIKVNFKIR HNIEDGSVQL ADHYQQNTPI GDGPVLLPDN HYLSTQSALS KDPNEKRDHM
	541	VLLEFVTAAG ITLGMDELYK HHHHHH
HBP	1	ATGCCAGAAC CATGTGAATT GGACGATGAA GATTTCAGAT GTGTATGTAA CTTCTCTGAA
	61	CCACAACCAG ATTGGTCTGA AGCTTTCCAA TGTGTTTCTG CTGTTGAAGT TGAAATCCAC
	121	GCTGGTGGTT TGAACTTGGA ACCATTCTTG AAGAGAGTTG ACGCTGACGC TGACCCAAGA
	181	CAATACGCTG ACACTGTTAA GGCTTTGAGA GTTAGAAGAT TGACTGTTGG TGCTGCTCAA
	241	GTTCCAGCTC AATTGTTGGT TGGTGCTTTG AGAGTTTTGG CTTACTCTAG ATTGAAGGAA
	301	TTGACTTTGG AAGATTTGAA GATCACTGGT ACTATGCCAC CATTGCCATT GGAAGCTACT
	361	GGTTTGGCTT TGTCTTCTTT GAGATTGAGA AACGTTTCTT GGGCTACTGG TAGATCTTGG
	421	TTGGCTGAAT TGCAACAATG GTTGAAGCCA GGTTTGAAGG TTTTGTCTAT CGCTCAAGCT
	481	CACTCTCCAG CTTTCTCTTG TGAACAAGTT AGAGCTTTCC CAGCTTTGAC TTCTTTGGAC
	541	TTGTCTGACA ACCCAGGTTT GGGTGAAAGA GGTTTGATGG CTGCTTTGTG TCCACACAAG
	601	TTCCCAGCTA TCCAAAACTT GGCTTTGAGA AACACTGGTA TGGAAACTCC AACTGGTGTT
	661	TGTGCTGCTT TGGCTGCTGC TGGTGTTCAA CCACACTCTT TGGACTTGTC TCACAACTCT
	721	TTGAGAGCTA CTGTTAACCC ATCTGCTCCA AGATGTATGT GGTCTTCTGC TTTGAACTCT
	781	TTGAACTTGT CTTTCGCTGG TTTGGAACAA GTTCCAAAGG GTTTGCCAGC TAAGTTGAGA
	841	GTTTTGGACT TGTCTTGTAA CAGATTGAAC AGAGCTCCAC AACCAGATGA ATTGCCAGAA
	901	GTTGACAACT TGACTTTGGA CGGTAACCCA TTCTTGGTTC CAGGTACTGC TGGTGGTGGT
	961	GGTGGTGGTG TTTCTAAGGG TGAAGAATTG TTCACTGGTG TTGTTCCAAT CTTGGTTGAA
	1021	TTGGACGGTG ACGTTAACGG TCACAAGTTC TCTGTTTCTG GTGAAGGTGA AGGTGACGCT
	1081	ACTTACGGTA AGTTGACTTT GAAGTTCATC TGTACTACTG GTAAGTTGCC AGTTCCATGG
	1141	CCAACTTTGG TTACTACTTT GACTTACGGT GTTCAATGTT TCTCTAGATA CCCAGACCAC
	1201	ATGAAGCAAC ACGACTTCTT CAAGTCTGCT ATGCCAGAAG GTTACGTTCA AGAAAGAACT
	1261	ATCTTCTTCA AGGACGACGG TAACTACAAG ACTAGAGCTG AAGTTAAGTT CGAAGGTGAC
	1321	ACTTTGGTTA ACAGAATCGA ATTGAAGGGT ATCGACTTCA AGGAAGACGG TAACATCTTG
	1381	GGTCACAAGT TGGAATACAA CTACAACTCT CACAACGTTT ACATCATGGC TGACAAGCAA
	1441	AAGAACGGTA TCAAGGTTAA CTTCAAGATC AGACACAACA TCGAAGACGG TTCTGTTCAA
	1501	TTGGCTGACC ACTACCAACA AAACACTCCA ATCGGTGACG GTCCAGTTTT GTTGCCAGAC
	1561	AACCACTACT TGTCTACTCA ATCTGCTTTG TCTAAGGACC CAAACGAAAA GAGAGACCAC
	1621	ATGGTTTTGT TGGAATTCGT TACTGCTGCT GGTATCACTT TGGGTATGGA CGAATTGTAC
	1681	AAGCACCACC ACCACCACCA CTAA

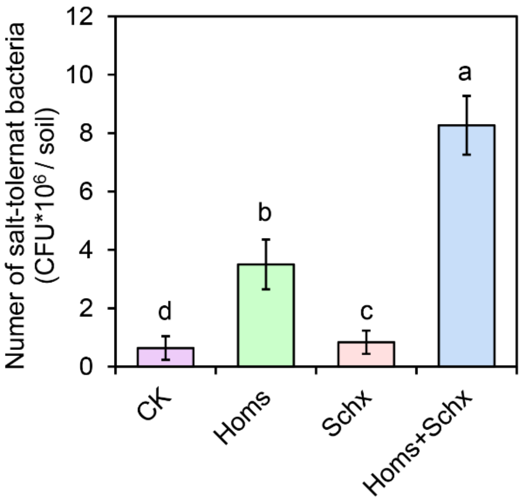

Figure S1 The number of halotolerant bacteria in the wheat rhizosphere soil after 30 days of cultivation. The different letters indicate significant difference between the groups (P<0.05).

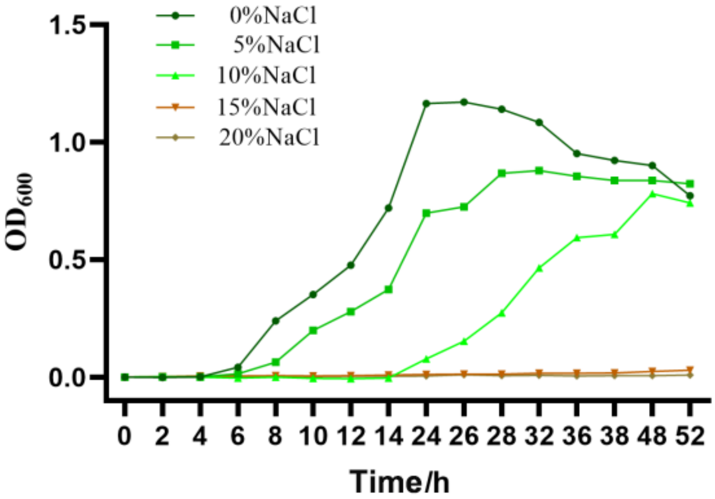

Figure S2 Growth curves of the Homs cells under different NaCl concentrations (0%, 5%, 10%, 15%, and 20%, w/v). Bacterial growth was monitored by OD_{600} over 52 h of incubation at 30 °C. Data are presented as means ± SD (n = 3). The results demonstrate that strain NK9104 is capable of robust growth across a wide salinity range, including salt-free conditions, confirming its halotolerant nature and supporting its application in the low-salinity soil environment (0.6% NaCl) used in this study.

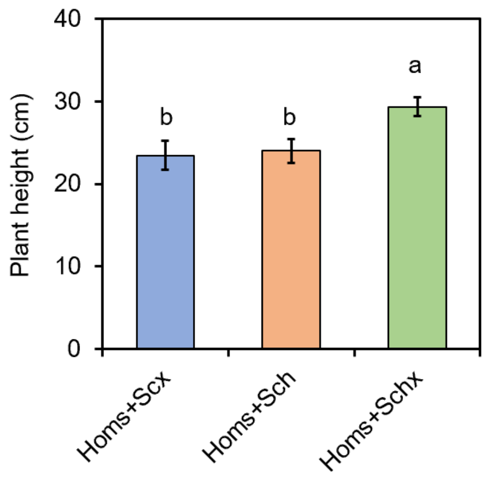

Figure S3 The effect of Homs+Scx, Homs+Sch and Homs+Schx on the tomato height under saline-alkali stress after 30 days of cultivation. The different letters indicate significant difference between the groups, while the identical letters indicate no significant difference ($P < 0.05$).
